# Performance of Toluene Removal in a Nonthermal Plasma Catalysis System over Flake-Like HZSM-5 Zeolite with Tunable Pore Size and Evaluation of Its Byproducts

**DOI:** 10.3390/nano9020290

**Published:** 2019-02-19

**Authors:** Weicheng Xu, Kaichun Lin, Daiqi Ye, Xueding Jiang, Junxing Liu, Yangda Chen

**Affiliations:** 1 School of Environment and Chemical Engineering, Foshan University, Foshan 528000, China; weichengxu@fosu.edu.cn (W.X.); kaichunlin2019@hotmail.com (K.L.); jiangxueding@fosu.edu.cn (X.J.); junxingliu2019@hotmail.com (J.L.); 2 School of Environment and Energy, South China University of Technology, Guangzhou 510006, China; 3 CT Environmental Group Limited, Guangzhou 510006, China; cdyman@gmail.com

**Keywords:** HZSM-5, dielectric barrier discharge, toluene, pore, byproduct

## Abstract

In this study, a series of HZSM-5 catalysts were prepared by the chemical liquid-phase deposition method, and low concentration toluene degradation was carried out in an atmospheric pressure dielectric barrier discharge (DBD) reactor. The catalysts were characterized by X-ray powder diffraction (XRD), SEM, TEM, and N_2_ adsorption analysis techniques. In addition, several organic contaminants were used to evaluate the adsorption performance of the prepared catalysts, and the effect of pore size on the removal efficiency of toluene and byproduct formation was also investigated. The unmodified HZSM-5 zeolite (Z0) exhibited good performance in toluene removal and CO_2_ selectivity due to the diffusion resistance of ozone and the amounts of active species (OH• and O•). Meanwhile, the time of flight mass spectrometry (TOF-MS) result showed that there were more byproducts of the benzene ring in the gas phase under the action of small micropore size catalysts. Moreover, the surface byproducts were detected by gas chromatography–mass spectrometry (GC-MS).

## 1. Introduction

Volatile organic compounds (VOCs) are recognized as precursors for the formation of organic aerosols, ground ozone, and photochemical smog [1,2]. Their emissions have adverse effects on human health, such as dry cough, dizziness, headache, and cancer [3,4]. Therefore, researchers have made great efforts to investigate different technologies for VOC abatement, including adsorption [5], membrane separation [6], thermal combustion [7], photocatalysis [8], and catalytic oxidation [9]. However, traditional VOC control methods have technical and economic limitations, especially for the treatment of low concentrations of VOCs. 

In recent decades, non-thermal plasma (NTP) technology has attracted more and more attention due to its inexpensive and easy operation to remove diluted VOCs [10,11]. However, some shortcomings—such as poor product selectivity, low efficiency, and the formation of unwanted byproducts (other VOCs, aerosols, NO_x_, O_3_)—in an NTP-only system hinder the development of industrial VOC abatement [12,13]. To overcome these limitations, many studies attempt to combine plasma technology with suitable heterogeneous catalysts [14,15,16]. Previous studies have demonstrated the positive effects of a plasma catalytic system on various VOC pollutants such as formaldehyde [17], acetaldehyde [18], trichloroethylene [19], benzene [2], toluene [20,21], and chlorobenzene [22]. In the periodical process, the selection of suitable catalysts plays an important role in the plasma catalysis system. In recent years, various catalysts including MnO_x_/Al_2_O_3_, CeMn/TiO_2_, MnO_2_-CuO/TiO_2_, AgCu/HZSM-5, and Co/MCM-41 have been developed for VOC abatement [14,22,23,24,25]. Van Durme et al. [23] studied the oxidation of toluene adsorbed on MnO_2_-CuO/TiO_2_ catalyst in a post-plasma catalytic system and found that the combination of NTP with MnO_2_-CuO/TiO_2_ had great potential for toluene decomposition. 

Fan et al. [14] investigated the decomposition of low-concentration benzene, toluene and p-xylene (BTX mixture) by a positive corona discharge on MnO_x_/Al_2_O_3_ catalyst. They concluded that the increase of relative humidity (30–80%) had slightly positive and negative effects on toluene and benzene removal, respectively. However, to our knowledge, it is essential to develop efficient methods to identify the byproducts and reduce their formation. Thus, this was one of the main objectives of this study. Moreover, zeolite molecular sieves have good hydrophobicity, high toluene adsorption capacity, and are also beneficial for VOC decomposition because of the stability and characteristics of the pore system [26,27,28]. Nevertheless, the studies on the relation among pore structure, catalytic activities, and the generation of byproducts remain limited. 

In this study, toluene was used as a representative VOC because it is widely applied as a solvent in several processes such as pressing, printing, and petrochemical industries. Different pore structures of HZSM-5 were prepared by the chemical liquid-phase deposition method, and their adsorption capacity and the catalytic performance of adsorbed toluene were evaluated. Moreover, an in situ time of flight mass spectrometry (TOF-MS) and an ozone analyzer were used to determine the gas-phase VOCs and O_3_ byproduct. The relation between pore structure and the generation of byproducts was also explored. 

## 2. Experimental

### 2.1. Preparation

HZSM-5 zeolite samples were obtained from Nanjing XFNANO Materials Tech. Co. Ltd. (Nanjing, China). Tetraethyl orthosilicate (TEOS) and cyclohexane were purchased from Aladdin Chemistry Co. Ltd. (Shanghai, China) and were used as a precipitant and a solvent, respectively. All the reagents were of analytical grade and were used without further purification.

Different pores sizes of HZSM-5 were synthesized by the chemical liquid-phase deposition method with TEOS and cyclohexane as the precipitant and solvent, respectively. In a typical process, 1 g of HZSM-5 was dissolved in a mixed solution containing a certain amount of TEOS and 10 mL cyclohexane. The obtained solution was stirred for several hours at room temperature. Then the mixture was heated and dried under the irradiation of an infrared lamp. After cooling to ambient temperature, the samples were heated from room temperature to 550 °C in a muffle furnace at the heating rate of 2 °C/min. After calcination at 550 °C for 4 h, the catalysts were naturally cooled to room temperature. 

According to the different amounts of TEOS, the samples were recorded as Z0, Z1, Z2, Z3, and Z4. Detailed information is displayed in Table 1.

### 2.2. Characterization 

The X-ray powder diffraction (XRD) measurements of the prepared catalysts were carried out on a Bruker D8-ADVANCE X-ray diffractometer (Bruker Axs Inc. Germany) with Cu Kα radiation (k = 0.15418 nm, 40 kV, 40 mA). The shape and morphology of the catalysts were recorded through a field emission scanning electron microscope (FE-SEM, Hitachi S–4800, Tokyo, Japan) and a transmission electron microscope (TEM, JEOL JEM-4000EX, Tokyo, Japan). The surface area was evaluated by the Brunauer–Emmet–Teller (Micromeritics TriStar II 3020, Micromeritics Instrument Corporation, Norcross, GA, USA) method, and the pore size distribution, average pore diameter, and total pore volume were measured by the Barrett-Joyner-Halenda (BJH) method. 

### 2.3. Experimental Setup

**Figure 1:**
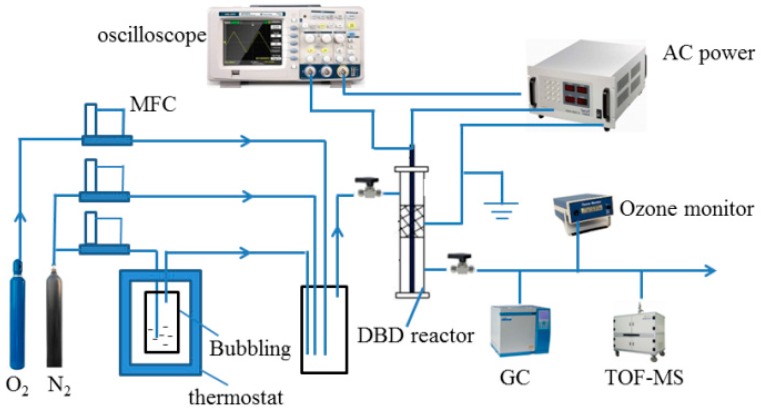
The schematic diagram of the experimental device.

The schematic diagram of the experimental device is shown in Figure 1, and it consisted of a bubbling system, an NTP-catalysis reactor, and a gas detection system. Toluene was evaporated by bubbling N_2_. The diluted toluene was mixed with N_2_ and O_2_ (N_2_:O_2_ (v:v) = 4:1) to achieve the concentration of 100 ppm in the mixing bottle. The dielectric barrier discharge (DBD) reactor was a wire-cylinder type made of quartz tube (outer diameter: 8 mm, length: 180 mm, wall thickness: 1 mm). A nickel rod with a diameter of 2 mm was placed at the reactor center as the high-voltage electrode. The grounding electrode was coated with a layer of copper wire outside the quartz glass tube. In this experiment, a high-voltage AC power supply (CTP-2000K, Su peak voltage man, Nanjing Suman Plasma Technology Co. Ltd., Nanjing, China) was used to generate an 8-kV discharge with a fixed frequency of 1.45 kHz. The discharge parameters were detected by oscilloscope (TDS1002, Tektronix, Beaverton, OR, USA). In the process of adsorption and catalysis, 150 mg catalyst was put into quartz tube at the airflow rate of 100 mL·min^−1^. In addition, the concentrations of toluene, CO_2,_ and CO in export gases were determined by gas chromatography (GC-2014C, Shimadzu, Japan). The gas-phase organic byproducts were detected by time of flight mass spectrometry (TOF-MS, SPIMS 1000, Hexin Mass Spectrometry Co. Ltd., Guangzhou, China), and the ozone (O_3_) concentration was determined by an O_3_ monitor (IDEAL-2000, Zibo Ideal Measurement and Control Technology Co. Ltd., Zibo, China). The residues of the catalyst were detected by gas chromatography–mass spectrometry (GCMS-QP2010 Ultra, Shimadzu, Japan). Toluene removal efficiency and carbon balance were defined as follows:

(1) $$\;\eta \;=\;\frac{C_{0}\;-C}{C_{0}}.\;$$

(2) $$\mathrm{Carbon}\;\mathrm{balance}=\frac{\left[CO_{2}]+[CO\right]}{7(C_{0}-C)}\times 100\%$$

where C_0_ and C were the inlet and outlet concentration of toluene; [CO] and [CO_2_] were the CO and CO_2_ concentrations in the gas stream during the plasma catalytic process.

## 3. Results and Discussion

**Figure 2:**
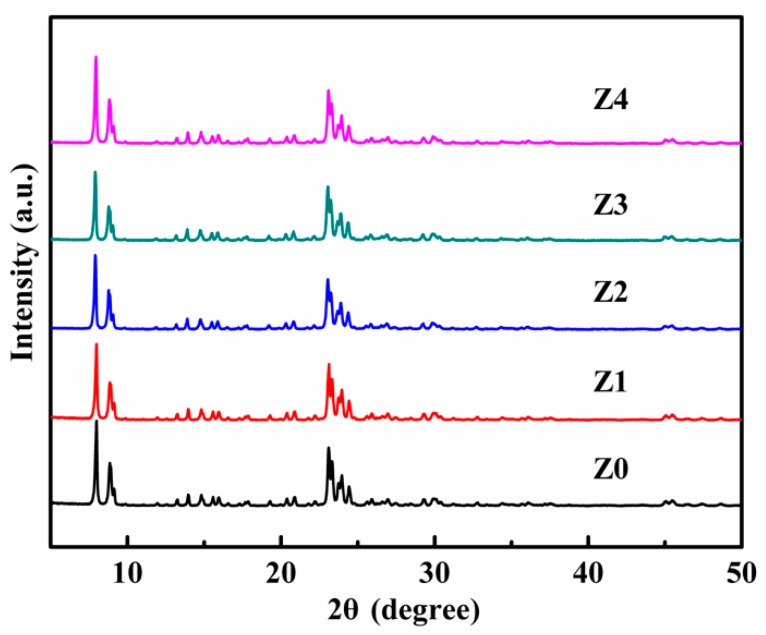
X-ray powder diffraction (XRD) spectra of the as-prepared catalysts.

Figure 2 shows the XRD results of the catalysts (Z0, Z1, Z2, Z3, and Z4). The intensive and sharp diffractions at 2θ = 7.9°, 8.8°, 23.9°, and 24.3° could be indexed by ZSM-5 (JCPDS42-0024) with a well-resolved mordenite framework inverted (MFI)structure [29]. Meanwhile, the result revealed that the synthetic method had no effect on the crystal structure of ZSM-5. 

**Figure 3:**
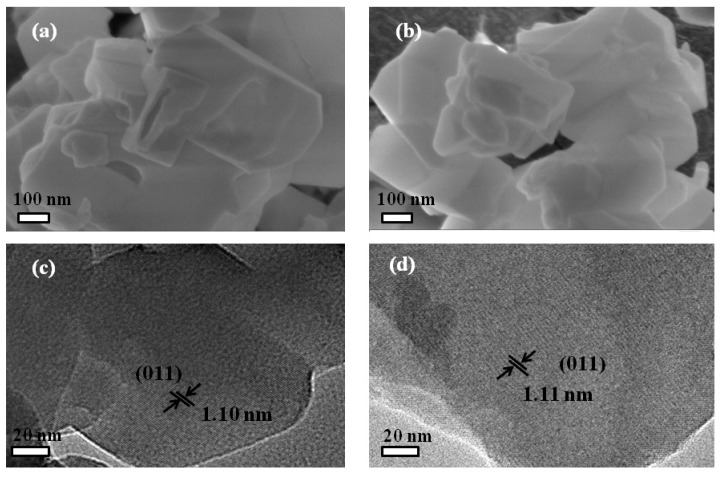
SEM images of (**a**) Z0, (**b**) Z4, and TEM images of (**c**) Z0, (**d**) Z4.

Figure 3 displays the SEM and TEM images of Z0 and Z4 samples. As shown in Figure 3a,b, two samples with flake morphology contained aggregations of nanoparticles with a relatively uniform size, indicating that the modification of the pore structure had little effect on their morphology. HRTEM images (Figure 3c,d) showed that the lattice fringes with d-spacing of about 1.10 nm corresponded well to the (011) lattice plane of ZSM-5 (JCPDS No. 42-0024), further confirming that the prepared nanomaterials also had an MFI structure [30].

**Figure 4:**
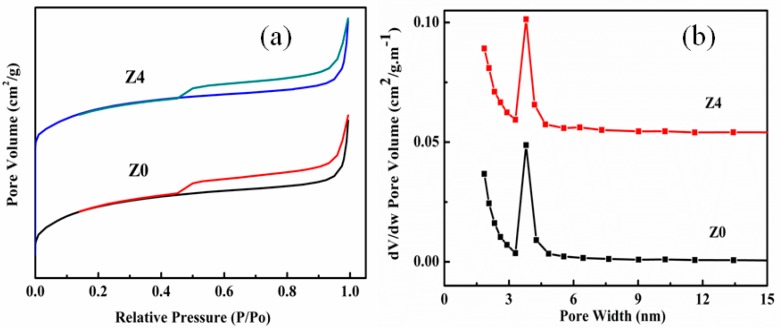
(**a**) N_2_ adsorption-desorption isotherms of Z0 and Z4, (**b**) N_2_ BJH desorption mesopore size distribution for Z0 and Z4.

Figure 4 shows the nitrogen adsorption-desorption isotherms of Z0 and Z4 sample. As shown in Figure 4, two samples exhibited type I isotherm according to International Union of Pure and Applied Chemistry (IUPAC) classification, indicating the characteristic of microporous solids [30]. After the treatment by the chemical liquid-phase deposition method, the isotherms had no significant change. The physicochemical properties of all catalysts are listed in Table 2. The slightly decreased BET surface area (S_BET_) and micropore specific surface areas (S_mic_) could be observed in the samples synthesized by the chemical liquid-phase deposition method. This phenomenon might be attributed to the effect of the precipitant and might affect the subsequent catalytic activity and the formation of byproducts. 

**Figure 5:**
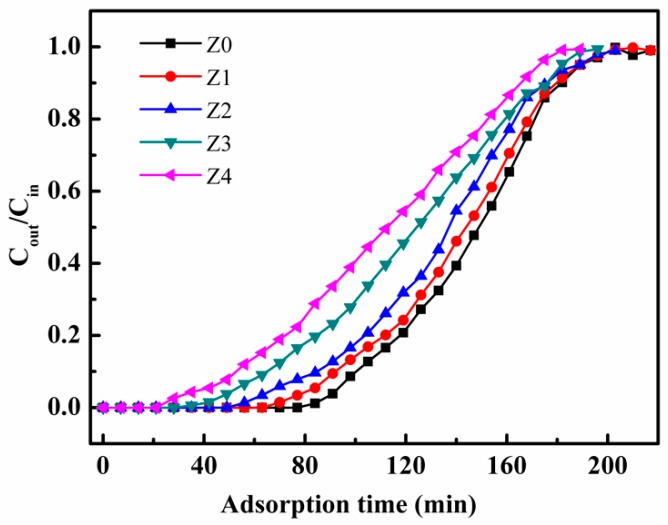
The breakthrough curves of toluene adsorption for different catalysts.

The adsorption curves of prepared samples are displayed in Figure 5. Obviously, the breakthrough time of Z0 was about 3 times higher than that of Z4, and the adsorption capacity of toluene decreased from 39.7 to 30.32 mg/g with the increase of the TEOS amount from 0 to 2.4 mL. This could be attributed to the fact that the S_BET_ and pore size of as-prepared catalysts gradually decreased with the increase of the precipitant concentration. Moreover, the channel dimension of pure Z0 was similar to the kinetic diameter of toluene; the mass transfer process of toluene molecules in HZSM-5 was susceptible to the change of pore size. As the pore size decreased, gas diffusion resistance increased, and the driving force of toluene adsorption became more negative in the adsorption site of HZSM-5.

In order to further investigate the effect of pore size on the adsorption potential, the adsorption experiments of p-xylene and m-xylene on the prepared catalysts were also carried out. As shown in Table 3, the adsorption capacity of p-xylene on Z0 was 34.23 mg/g, which was slightly higher than that obtained on Z4 (30.17 mg/g). The adsorption capacity of m-xylene on Z0 was 10.58 mg/g, which was much larger than that on Z4 (1.10 mg/g). This result indicated that the adsorption of p-xylene on the prepared catalysts was significantly higher than the adsorption of m-xylene, and this could be attributed to the effect of size. The channel dimensions of as-prepared HZSM-5 were similar to the kinetic diameters of toluene and p-xylene [31,32,33], and micropore diffusion might affect the overall reaction rate and adsorption capacity [34].

**Figure 6:**
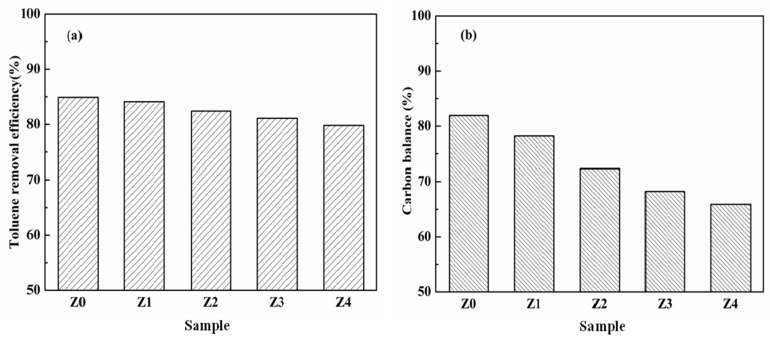
(**a**) Toluene removal efficiency and (**b**) carbon balance of the as-prepared catalysts.

Figure 6 shows the toluene removal efficiency of toluene and the carbon balance of the prepared catalysts in the plasma catalytic systems. As expected, the conversion of toluene decreased slightly from 84.9% (Z0 sample) to 79.8% (Z4 sample). The similar crystal structure and surface properties of the catalysts were directly proportional to the amount and energy of high-energy electrons in plasma catalysis [26], so the removal efficiency of toluene by the catalysts showed little difference. However, the carbon balance decreased significantly from 81.9% in Z0 to 65.8% in Z4. This could be attributed to the adsorption capacity and ozone utilization [35]. Moreover, the acidity of zeolite might have an effect not only on the removal efficiency, but also on the adsorption properties of the zeolite [36].

**Figure 7:**
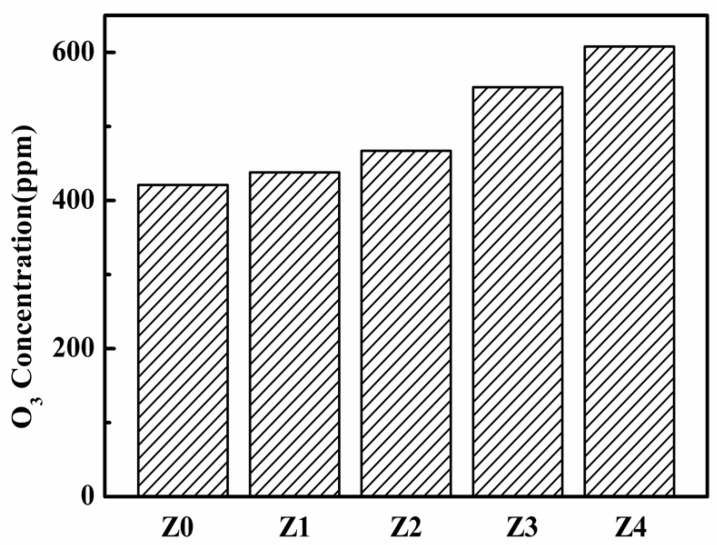
Ozone concentrations of the prepared catalysts.

The ozone concentrations of different catalysts are shown in Figure 7. In the plasma catalytic process, it was believed that the destruction of VOCs is mainly due to ozone, which was acting either directly or indirectly via adsorption or decomposition on catalyst surfaces, thereby creating active species to oxidize adsorbed VOCs [37,38]. As shown in Figure 7, the ozone concentration of Z4 was 1.5 times higher than that of Z0. This could be ascribed to the fact that the diffusion resistance of ozone in the pore tunnel of catalysts was enhanced with the decrease of pore size, and less ozone migrated toward the surface of the catalysts to decompose toluene. This result was well correlated with the result in Figure 6b, which showed that higher CO_x_ concentrations were observed with Z0 than those with Z4.

**Figure 8:**
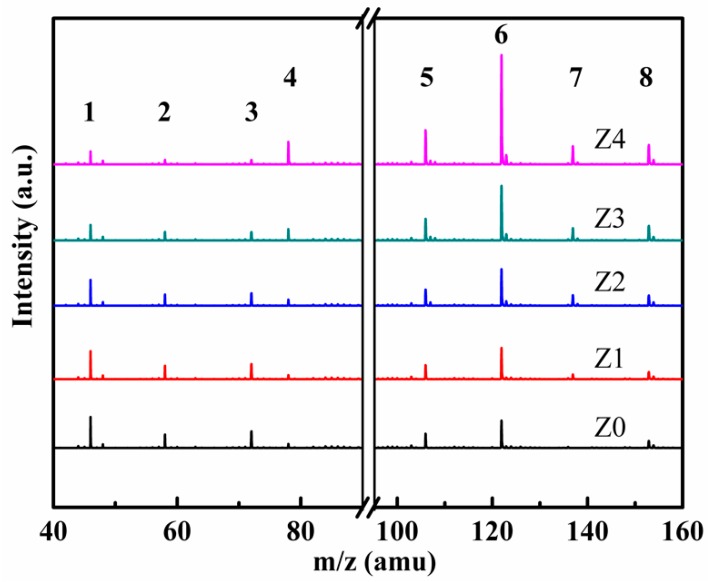
Time of flight mass spectrometry (TOF-MS) spectrum of organic byproducts in the gas phase over a series of HZSM catalysts in a plasma catalysis system: 1. formic acid, 2. oxalaldehyde, 3. methylglyoxal, 4. benzene, 5. benzaldehyde, 6. benzoic acid, 7. nitrotoluene, 8. nitrobenzyl alcohol.

Figure 8 demonstrates the gas-phase organic byproducts of various catalysts in plasma catalysis. As shown in Figure 8, the intensity of m/z was 78, 106, 122, 137, and 153, increasing from Z0 to Z4. The result indicated that more benzene ring byproducts such as benzene, benzaldehyde, benzaldehyde, nitrotoluene, and nitrobenzyl alcohol were easily maintained in the gas phase in the catalysts with smaller pore diameters. On the contrary, more small gas-phase compounds such as formic acid, oxalaldehyde, and methylglyoxal could be found in the catalysts with bigger pore diameters during plasma catalysis. This result suggested that the amounts of oxygen species (OH• and O•) were greater on large-pore-size HZSM-5 than those on small-pore-size HZSM-5, and the active oxygen species decomposed from O_3_ to the oxygen vacancy in the catalysts were in charge of toluene deep oxidation [39]. Therefore, toluene could be deeply oxidized into small byproducts in the Z0 sample.

**Figure 9:**
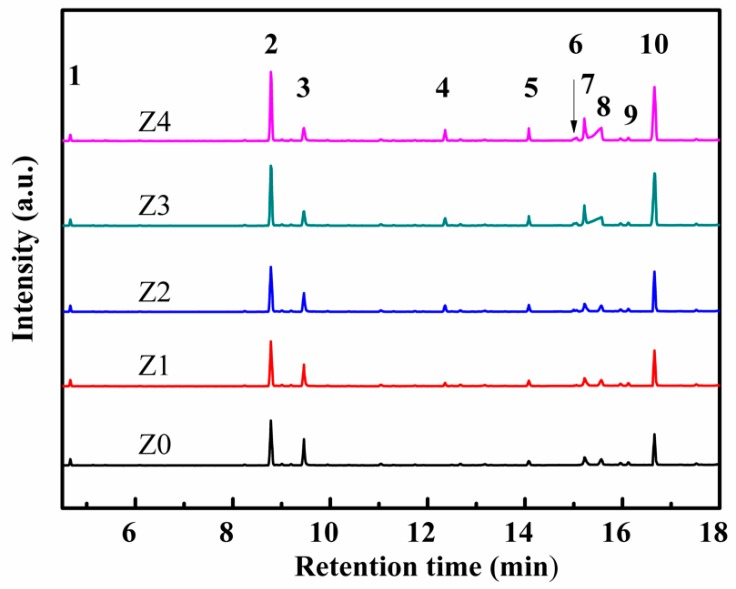
GC-MS spectrum of organic byproducts of toluene degradation over different catalysts by plasma catalysis: 1. n-octane, 2. benzaldehyde, 3. phenol, 4. benzyl formate, 5. o-nitrophenol, 6. o-nitrotoluene, 7. o-nitrobenzene methanol, 8. benzoic acid, 9. n-dodecane, 10. nitrotoluene.

The catalyst surface byproducts identified by GC-MS were shown in Figure 9. Almost 10 byproducts could be found in the surface of catalysts after toluene degradation. Most of these byproducts were benzene ring products, which might affect the deactivation of the catalysts [15]. Compared with the Z4 catalyst with a small pore size, byproducts with a large pore size—e.g., the Z0 sample—had fewer byproducts due to more active species available for the reaction and reduction of byproducts. This result was consistent with the analysis results of ozone and gas-phase byproducts above. Meanwhile, several nitro-containing byproducts were found on catalyst surfaces after plasma catalysis. O-nitrotoluene appeared due to the reaction of toluene with radicals, and the formation of o-nitrophenol might be attributed to the reaction between phenol and excited NO_2_ [40,41]. More interestingly, the amount of phenol increased slightly with the increase of the pore size of HZSM-5. This might be due to the amount of toluene adsorption, and because more adsorbed toluene molecules were oxidized on the surfaces of catalysts and more phenol was formed. 

## 4. Conclusions

In conclusion, a simple chemical liquid-phase deposition method was established using TEOS as a precipitant for the synthesis of different pore structures of HZSM-5, and toluene oxidation was evaluated under plasma catalysis. It was indicated that with the decline of the S_BET_ (from 366 m^2^/g to 341 m^2^/g) and the micropore size (from 0.533 nm to 0.522 nm), toluene adsorption and catalytic performance gradually decreased. The decreased removal efficiency could be attributed to the enhanced diffusion resistance of ozone and the reduction of active species (OH• and O•). Moreover, TOF-MS results showed that more benzene ring byproducts were easily maintained in the gas phase in the catalysts with smaller pore diameters. Almost 10 byproducts could be found on the surface of catalysts after plasma catalysis, and benzene ring byproducts might affect the deactivation of the catalysts. GC-MS result also showed that the amount of phenol increased slightly with the increase of pore size of HZSM-5 due to the oxidation of more toluene molecules adsorbed on the surface of HZSM-5 with a large pore size.

## Figures and Tables

**Table 1:** The component ratios of as-prepared catalysts.

Sample	HZSM-5	Cyclohexane	TEOS
Z0	pure HZSM-5	0	0
Z1	1g	10 mL	0.2 mL
Z2	1g	10 mL	0.8 mL
Z3	1g	10 mL	1.6 mL
Z4	1g	10 mL	2.4 mL

**Table 2:** Surface properties of the catalysts.

Sample	S_BET_ (m^2^/g)	S_mic_ (m^2^/g)	V_tot_ (cm^3^/g)	V_mic_ (cm^3^/g)	D_mic_ (nm)
Z0	366	330	0.180	0.145	0.533
Z1	360	328	0.179	0.145	0.531
Z2	354	325	0.178	0.146	0.526
Z3	349	318	0.174	0.141	0.523
Z4	341	313	0.175	0.140	0.522

**Table 3:** The adsorption capacity of as-prepared catalysts for different contaminants.

Sample	Equilibrium Adsorption Capacity (mg/g)		
	Toluene	p-xylene	m-xylene
Z0	39.70	34.23	10.58
Z1	38.92	34.15	9.17
Z2	36.53	34.02	6.96
Z3	33.31	31.42	3.84
Z4	30.32	30.17	1.10
